# Academic Schedule and Day-to-Day Variations in Sedentary Behavior and Physical Activity of University Students

**DOI:** 10.3390/ijerph17082810

**Published:** 2020-04-19

**Authors:** H. Q. Chim, Mirjam G. A. oude Egbrink, Pascal W. M. Van Gerven, Renate H. M. de Groot, Bjorn Winkens, Hans H. C. M. Savelberg

**Affiliations:** 1 Department of Nutrition and Movement Sciences, School of Health Professions Education (SHE), Faculty of Health, Medicine and Life Sciences (FHML), Maastricht University, 6200 MD Maastricht, The Netherlands; 2 Department of Physiology, SHE, FHML, Maastricht University, 6200 MD Maastricht, The Netherlands; m.oudeegbrink@maastrichtuniversity.nl; 3 Department of Educational Development and Research, SHE, FHML, Maastricht University, 6200 MD Maastricht, The Netherlands; p.vangerven@maastrichtuniversity.nl; 4 Department of Complex Genetics, School of Nutrition and Translational Research in Metabolism (NUTRIM), FHML, Maastricht University, 6200 MD Maastricht, The Netherlands; Renate.deGroot@ou.nl; 5 Faculty of Educational Sciences, Open University of The Netherlands, 6419 AT Heerlen, The Netherlands; 6 Department of Methodology and Statistics, Care and Public Health Research Institute (CAPHRI), FHML, Maastricht University, 6200 MD Maastricht, The Netherlands; bjorn.winkens@maastrichtuniversity.nl; 7 Department of Nutrition and Movement Sciences, SHE, NUTRIM, FHML, Maastricht University, 6200 MD Maastricht, The Netherlands; hans.savelberg@maastrichtuniversity.nl

**Keywords:** activity monitoring, education, lifestyle

## Abstract

Students starting at university tend to adopt unhealthy behaviors. With students expected to sit during classes, their academic schedule may be responsible for their activity patterns. The aim of the current study was to investigate the relationship between university students’ academic schedule and day-to-day variations in sedentary behavior (SB) and physical activity (PA). The activity of 317 first-year undergraduate students (mean age 19.6 ± 1.4 years, 69.4% female, 30.0% male, and 0.6% other) was measured with the activPAL3™ triaxial monitor for seven consecutive days. Each class hour was found to be associated with 9.0 additional minutes of SB (95% CI [4.9, 13.1]), 54 additional seconds of moderate-to-vigorous PA (MVPA; 95% CI [12, 96]), and 12.2 min less time in bed (95% CI [−16.6, −7.8]). Active SB ratio (total duration of SB bouts < 30 min divided by total SB duration) decreased by 0.011 per hour of class scheduled for the students (95% CI [−0.016, −0.006]). Light PA (LPA) was not significantly associated with class duration. Students tend to cycle more on days with classes. Seated transportation was not significantly related to whether the students had classes or not. Overall, the academic schedule is associated with SB and PA in students.

## 1. Introduction

Sedentary behavior (SB) covers all activities carried out in a sitting, reclining, or lying posture (excluding sleep), while expending energy of up to 1.5 metabolic equivalents (METs; METs are multiples of oxygen consumption during seated rest [1], with one MET being approximately 3.5 mL/min/kg [2]). Reviews of longitudinal studies have consistently shown that SB is associated with cardiovascular diseases [3,4], type 2 diabetes [3,4,5,6,7], and all-cause mortality [3,5,6,8]. For example, sedentarily watching television for at least two hours each day was associated with 13% higher risk of all-cause mortality [3] and 5% higher risk of cardiovascular diseases [8]. Various governmental bodies have recommended minimizing SB [9], with numerous studies proposing that people replace or break up SB with light physical activity (LPA) or moderate-to-vigorous physical activity (MVPA) [10,11,12,13]. Physical activity (PA) is defined as all bodily movements carried out by the skeletal muscles that increase energy expenditure [14], with LPA covering >1.5 to <3.0 METs, and MVPA starting from 3.0 METs [2]. Years of research have supported the health benefits of PA, serving as primary and secondary preventions of obesity, cardiovascular diseases, diabetes, hypertension, depression, and premature death [15,16]. Nevertheless, PA and SB are not completely dependent on each other [17], with the metabolic risks associated with prolonged SB being present even in those who perform MVPA regularly [18].

Unfortunately, students starting their higher education tend to adopt unhealthy behaviors. For example, these students spend less time on sports (because they no longer participate in sports clubs or they lack time) and active transportation (because they live on or nearby campus) compared to when they were in secondary education [19]. Although students spend less time on certain SB, such as watching TV and playing computer games, other SB such as internet use and studying tend to increase [19]. These unhealthy behaviors, i.e., reduced PA and increased SB, are associated with an increased risk of weight gain [20,21,22,23]. This is further supported by a meta-analysis of longitudinal studies that highlighted the high prevalence of weight gain in first-year undergraduate students, with almost two-thirds of students gaining weight at a rate that is much faster than the general population [24]. The implications of this should be taken seriously because more SB and less PA in adolescence and early adulthood is associated with a higher body mass index (BMI) in later adulthood [25,26,27]. Thus, there is a need to understand the PA and SB of these students in relation to the academic setting that they are embedded in.

Owen et al. stress that the behavior setting is important when trying to understand the determinants of PA and SB, because behaviors are shaped by the attributes and social frames of the setting [28]. In this study, the behavior setting would be the college/university environment, to which students have reported that their SB and PA were influenced by their social environment, their physical environment, the macro environment (e.g., school policy, media, and advertising), and university characteristics [29]. Upon starting college/university, other than increased internet use and studying while being sedentary [19], it is also the current norm for students to sit throughout their scheduled classes. We suspect that the imposed sitting during the scheduled classes plays a role in the students’ day-to-day physical activity. Longer class durations impose longer SB on students, leaving less time for PA. However, we do not expect the students’ activities to be homogeneous from day to day. For example, the students may be more active towards the end of the week than at the start of the week. Therefore, the aim of this study was to explore the association between the academic schedule and the students’ day-to-day variations in activity patterns. It is hypothesized that attending classes is related to the students’ day-to-day activity patterns, with longer class durations associated with more SB and less PA. To attend classes, the students would need to travel to the campus, with a choice of commuting actively (e.g., walking or cycling) or passively (e.g., by car or public transport). Therefore, we also explored whether the use of active and passive transportation is related to the students’ academic schedule. We did not expect the duration or type of transportation to change with class duration, but hypothesized that students would use transportation on days with scheduled classes (to commute to campus) rather than on days without classes.

## 2. Materials and Methods

### 2.1. Study Design and Setting

This study utilized a cross-sectional, observational design. Recruitment was conducted from May 2017 until April 2018 at Maastricht University with academic schedules varying from zero to eight hours of classes per day. The reporting of this study followed the Strengthening the Reporting of Observational Studies in Epidemiology (STROBE) statement [30]. This study was assessed by the Medical Ethics Committee of Maastricht University Medical Center+ and Maastricht University (reference number METC17-4-072), with the Medical Research Involving Human Subjects Act (WMO) was not applicable for this study.

### 2.2. Participants

First-year undergraduate students at Maastricht University were recruited using non-probability, convenience sampling. We focused on first-year students to acquire an optimally homogeneous study sample. Exclusion criteria were not being a first-year student, or having musculoskeletal discomfort or other pathologies that would influence physical behaviors.

### 2.3. Materials

The participants completed a demographics questionnaire, consisting of questions on class duration scheduled during the measurement week, age, height, weight, gender, commuting to university, part-time employment status, residential area, degree program, living situation, highest parental education achievement, gym/sports club membership status, smoking status, and alcohol consumption.

The activPAL3™ (PAL Technologies Ltd., Glasgow, UK) is recommended for field-based monitoring of free-living activities due to its ability to detect limb position and acceleration [31]. The activPAL3™ has been shown to be a reliable and valid measure of physical activity and sedentary behavior for the adult population [32]. In this study, the activPAL3™ was used to identify time in bed, lying/sitting, standing, stepping, cycling, and seated transportation [33]. The activPAL3™ was programmed to log activity for seven continuous days, waterproofed with a nitrile sleeve and Tegaderm transparent film, before attachment on the participant with another 10 × 10 cm Tegaderm film. A 5 × 5 cm compression bandage between the activPAL3™ and the skin provided gentler skin contact. Wearing the small and lightweight activPAL3™ (35 × 53 × 7 mm, weighing 15 g) was assumed to not affect the daily behavior of the students, as shown by studies where no evidence was found for reactivity towards wearing motion sensors or pedometers [34,35]. As a supplementary check for errors detected by the activPAL3™, participants completed an online diary, based on the International PA Questionnaire Short Form modified for daily use [36].

### 2.4. Procedure

The researcher met with individual participants in private rooms equipped with a laptop or tablet. Verbal and written information were given to the participants, including reminders that participation was voluntary, that they had the right to withdraw at any time without an explanation, and that their personal data would be anonymized and kept private and confidential. The participants could voice concerns or questions at any time during the study. Verbal and written informed consent were obtained from each participant.

After completing the demographic questionnaire, the activPAL3™ was attached to the middle-anterior of the participants’ right thigh and they were given access to their online diary. The meeting took approximately 15 min.

### 2.5. Activity Variables

Data recorded on the activPAL3™ was processed using the CREA algorithm of the activPAL’s data processing software, PALbatch (Version 8.10.9.43, PAL Technologies Ltd., Glasgow, UK). PALbatch’s setting was set to the most conservative 24-h wear time protocol for validating a day. When non-wear duration accumulated to four continuous hours or more, the CREA algorithm would classify this as a non-valid day. Each valid day started at midnight and lasted 24 h.

The extracted sitting time represented SB duration. Although various parties have recommended reducing SB as much as possible, the complete eradication of SB from daily life is inconceivable. Taking into consideration the recommendation to interrupt SB every 30 min [37], the variable “active SB ratio”, defined in Equation (1) was created. The higher the active SB ratio, the less detrimental the effects of total duration of SB is expected to be.

(1) $$\text{Active SB ratio}=\frac{total\;duration\;of\;SB\;bouts\;lasting\leq 30\;minutes}{total\;duration\;of\;SB}$$

MVPA duration was taken from stepping time with a cadence of ≥100 steps/min [38]. LPA was inferred from the definitions of MVPA and SB, covering all PA below the threshold of MVPA and above the threshold of SB. Therefore, LPA duration comprised of standing and stepping time with a cadence of <100 steps/minute. Time in bed is a combination of primary (e.g., nighttime sleep) and secondary lying (e.g., daytime naps) time during each day, estimated from proprietary algorithms that identified time in bed. Cycling and seated transportation were reported as an output variable by the CREA algorithm. Cycling is part of overall PA while seated transportation is a subsection of SB. We do not expect cycling and seated transportation to increase or decrease with class duration. Instead, the students are expected to commute to university, attend class(es), and then commute home. Therefore, cycling and seated transportation were analyzed with the binary variables of having classes and not having classes.

### 2.6. Statistical Analyses

All statistical analyses were performed using IBM SPSS Statistics for Windows (version 25.0, Armonk, NY, USA). Two-sided *p*-values ≤ 0.05 were considered statistically significant. Linearity assumption for numerical explanatory variables was checked using scatterplots. Due to the relatively large sample size, possible violations to the normality assumptions are not a concern [39,40], but nonetheless this was checked with histograms and qq-plots. The demographical information of the sample was summarized using means (M), standard deviations (SD), frequencies (N), and percentages (%).

Weekends were excluded from the analyses for two reasons: because classes were only scheduled on weekdays and because weekday activity patterns have been shown to be different from weekend activities [41]. Pearson’s correlation was used to analyze associations between the four activities (i.e., SB, LPA, MVPA, and time in bed) that constitute a 24-h day.

Marginal models were used as they incorporate all available data, account for correlations between repeated measures, and use a likelihood approach for missing outcome data (assuming missingness at random (MAR), that is the probability of missing only depends on observed, but not on unobserved variables). Missing outcome might occur, for example, when students remove the activPAL3™ before the scheduled date. Using logistic regression, if there were demographical variables related to the missing outcome data, these variables were added to the corresponding models to satisfy the MAR assumption of the model. As gender and body mass index (BMI) tend to covary with SB and PA [42,43,44,45,46], these variables were controlled for within each model. Fixed parts of the models contained day of the week (categorical) and class duration (numerical), where an unstructured covariance type was selected for the repeated measures within a student. To assess whether the association between class duration and the outcome depended on day of the week, a two-way interaction between day of the week and class duration was included in the model, together with the main effects. The likelihood ratio tests (based on maximum likelihood (ML) estimation) were used as the main check for whether these interactions improved model-fit. The Akaike’s Information (AIC) and Schwarz’s Bayesian Criteria (BIC) based on ML estimation were used to check the results. The AIC and BIC are goodness-of-fit measures, correcting for the number of parameters included in the model. The BIC is a more conservative version, used for large sample sizes. Smaller values of AIC and BIC indicate better-fitting models [47]. In case the interaction was not significant, it was removed from the model and main effects were reported. If the interaction was significant, the class duration effect was reported for each day of the week separately. For cycling and seated transportation, marginal models were assessed in the same way as for the models described before, except that class duration was dichotomized to the binary variables of days having classes and days having no classes. The results of the final models were reported, i.e., estimated coefficients of the explanatory variables with their corresponding 95% confidence intervals (CI) and *p*-values based on restricted maximum likelihood (REML) estimation. If there was a main effect of the weekday, pairwise comparisons of weekdays that were significantly different from each other were reported, with Cohen’s *d* calculated based on paired data, that is, mean of difference scores divided by standard deviation of difference scores. For comparison reasons, the absolute value of Cohen’s *d* was presented.

## 3. Results

### 3.1. Descriptive Statistics

Of the 335 participants’ data, three were excluded due to technical errors, eight were excluded because they were not first-year students, and seven were excluded due to incomplete demographic questionnaires. The final sample consisted of 317 participants. Each day, three to nine participants temporarily removed the activPAL3™, resulting in non-wear duration. When non-wear duration accumulated to four continuous hours or more, the CREA algorithm classified this as a non-valid day for that student, resulting in three non-valid Fridays. The daily diaries did not serve as a good check for non-wear duration and non-valid days as compliancy for logging into the daily diary was very low, with 201 students having incomplete diaries. Non-wear duration and non-valid days were not significantly related to any of the demographical variables (*p*’s > 0.05).

The participants had a mean age of 19.6 years (SD = 1.4), mean height of 1.73 m (SD = 0.09), and mean weight of 65.5 kg (SD = 10.3). Using the World Health Organization’s classification of body mass index (BMI) [48], 6.3% of the participants were underweight, 83.9% were in the healthy range, and 9.8% were either overweight or obese. Detailed self-reported demographical characteristics of the sample can be found in Table 1.

Table 2 summarizes the behavioral characteristics of the sample. The correlations between the four activities that make up a 24-h day (i.e., SB, LPA, MVPA, and time in bed) are presented in the Appendix A Table A1. As these activities are constrained within the 24-h day, they do correlate with one another, albeit weakly (*r* < 0.05). Only time in bed correlated strongly with SB (ranging from *r* = −0.63 to −0.73, *p* < 0.001).

**Figure 1:**
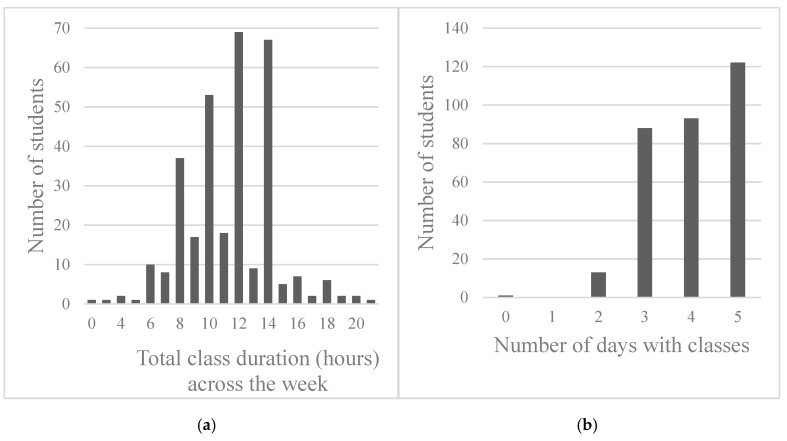
(**a**) Number of students across the total duration of classes (hours) scheduled across the week; (**b**) Number of students across the total number of class days scheduled across the week. Class duration (hours) was rounded up. For example, 1.5 h is rounded up to 2.0 h for illustrative purposes.

The students had 1.5 to 3.0 h of classes per day on average, amounting to between 8.0 and 14.0 h per week for most students (Figure 1a), with approximately 60% of the students sample having less than five class days per week (Figure 1b). The average SB/day spanned from 8 h 20 min to 8 h 58 min, with approximately half of the total SB duration spent in bouts of less than 30 min. The students spent on average 4 to 5 h/day in LPA, and approximately 30 min/day in MVPA. Time in bed (consisting of both primary and secondary lying time) lasted approximately 10 h/day, which contrasted with the students’ self-reported average (primary) sleep time of 7.5 h (SD = 3.7). In terms of daily commuting, the activPAL3™ detected that the students spent an average of 16 to 19 min cycling and 21 to 36 min in seated transportation per day. Although there may be other purposes for commuting, the students self-reported that they travel for an average of 18.6 min (SD = 21.7) to attend classes at university, with 95.6% of the sample (303 students) reporting that they commute to university by bike or by foot.

### 3.2. Association between Academic Schedule and Students’ Activity Levels

The likelihood ratio tests showed that the two-way interaction between weekdays and class duration did not improve the models significantly, which was confirmed by the lowest AIC and BIC produced for all models without any interactions (SB, χ^2^ (4) = 0.65; active SB ratio, (χ^2^ (4) = 3.74; LPA, χ^2^ (4) = 2.52; MVPA, χ^2^ (4) = 3.73; time in bed, χ^2^ (4) = 2.27; cycling, χ^2^ (4) = 2.67; seated transportation, χ^2^ (4) = 2.52; all *p*’s > 0.05; see Appendix A Table A2 for AIC and BIC values). Therefore, the models without any interactions were used, based on REML estimation, when interpreting the relationship between class duration and each activity across weekdays.

The marginal models, summarized in Table 3, revealed a significant main effect of class duration on SB (*p* < 0.001), active SB ratio (*p* < 0.001), MVPA (*p* = 0.010), and time in bed (*p* < 0.001). Each hour of class was associated with 9.0 additional minutes of SB (95% CI [4.9, 13.1]) and 54 additional seconds (0.9 min in Table 3; 95% CI [0.2, 1.6]) of MVPA, with 12.2 min less time in bed (95% CI [−16.6, −7.8]). Active SB ratio saw a drop by 0.011 (95% CI [−0.016, −0.006]) with each scheduled class hour. Time spent in LPA (95% CI [−1.6, 4.0], *p* = 0.413) was not significantly associated with class duration.

As shown in Table 4, on days with scheduled classes, students spent on average 4.0 min more on cycling (95% CI [1.9, 6.0], *p* < 0.001) compared to days with no classes. Duration of seated transportation was not significantly related to whether or not the students had classes (95% CI [−3.8, 7.1], *p* = 0.549).

### 3.3. Day-to-Day Variations of Students’ Activity Levels

There was also a significant main effect of weekdays on SB (*p* = 0.045), active SB ratio (*p* = 0.004), LPA (*p* < 0.001), and time in bed (*p* = 0.007), showing significant day-to-day changes in these activities. Table A3, Table A4, Table A5 and Table A6 in the Appendix A display the pairwise comparisons between weekdays.

After controlling for the effects of class duration, the students were most sedentary on Wednesdays (21.5 min more than Thursdays (95% CI [1.5, 41.6], *p* = 0.036, *d* = 0.10) and 29.3 min more than Fridays (95% CI [9.8, 48.7], *p* = 0.003, *d* = 0.21). They had the highest active SB ratio on Fridays (0.029 to 0.048 more compared to all weekdays, all *p*’s < 0.05, Cohen’s *d* ranging from 0.15 to 0.23). Furthermore, the students spent most time on LPA on Fridays (20.9 to 33.5 min more compared to Tuesdays, Wednesdays, and Thursdays, all *p*’s < 0.05, Cohen’s *d* ranging from 0.13–0.24), with the least amount of time on LPA on Mondays (17.1 to 40.2 min less than Wednesday, Thursdays, and Fridays, all *p*’s < 0.05, Cohen’s *d* ranging from 0.15 to 0.30). The students spent the most time in bed on Mondays (32.5 min more than Wednesdays (95% CI [13.0, 52.1], *p* = 0.001, *d* = 0.15) and 32.4 min more than Fridays (95% CI [9.7, 55.1], *p* = 0.005, *d* = 0.08)) and Tuesdays (23.4 min more than Wednesdays (95% CI [4.3, 42.6], *p* = 0.017, *d* = 0.12), and 23.3 min more than Fridays (95% CI [2.2, 44.3], *p* = 0.031, *d* = 0.06)).

Controlling for the effects of classes, the students’ cycling duration did not differ significantly between weekdays (*p* = 0.748). Duration of seated transportation differed significantly between weekdays (*p* = 0.001). Pairwise comparisons are shown in Table A7 of the Appendix A. Students spent 12.3 to 15.6 min more in seated transportation on Fridays compared to other weekdays, all *p*’s < 0.05, Cohen’s *d* ranging from 0.17 to 0.21.

## 4. Discussion

The current study explored the relationship between the academic schedule and day-to-day activity patterns of first-year undergraduate students at Maastricht University. Class duration was found to have a significant positive relationship with SB and MVPA, a significant negative relationship with active SB ratio and time in bed, but no significant relationship with LPA. On weekdays when classes were scheduled, the students spent more time cycling compared to weekdays without scheduled classes.

Overall, first-year students spent close to nine hours per day being sedentary during the weekdays. Using the activPAL3™, the current study revealed that the students were more sedentary than the median of five to six hours reported in previous studies employing self-reported measures [50], which is not unusual considering the tendency to underestimate one’s SB duration [51]. Importantly, every additional hour of class was found to be associated with nine more minutes of SB. With students attending 1.5 to 3.0 h of classes per day, the students are expected to engage in 14 to 27 additional minutes of SB on days with classes compared to days without classes. With class duration going up to eight hours per day, the students can accumulate up to 72 additional minutes of SB. The additional minutes of SB take away time that could have been spent on more active behaviors, such as participating in sports. However, when no classes were scheduled, the students were also rather sedentary. At the university where recruitment took place, students were expected to spend a considerable part of the week self-studying in preparation for their tutorial discussions (see Maastricht University’s problem-based learning educational model [52]), which may have contributed to their SB outside the classrooms. Similarly, students in another study report that at university, they engaged in more studying and internet use while being sedentary, while having less time to engage in sports or any sports clubs [19]. In the current study, approximately 30% of students report not engaging in any gym or sports clubs while 10% report having an inactive membership. To summarize, students spend close to nine hours per day being sedentary, with SB increasing further with class duration. This necessitates both the educational institution and the individual student to find ways of balancing education with an active, less sedentary lifestyle.

The results also show that class duration was negatively related to active SB ratio. This was expected, as an average class took one to two hours. This also suggests that outside of classrooms, students tend to engage in a higher frequency of short SB bouts, relative to their overall SB duration. The beneficial effects of taking frequent breaks from SB have been demonstrated in numerous experimental studies [53]. Educational institutions could introduce short, active breaks between classes, with LPA breaks as short as 10 min being sufficient to reduce the risks of metabolic syndromes [54].

Time in bed was rather long, ranging from 10 to almost 12 h per day. Time in bed in this study consists of both primary (i.e., nighttime sleep) and secondary lying time (e.g., daytime naps). The students reported that they sleep an average of only 7.5 h per night. It is important to note that time in bed estimated by limb position does not necessarily indicate restorative sleep. Time spend in bed while being awake is also considered SB. The blurry line that separates the thresholds of SB and time in bed could explain for the strong negative correlation between time in bed and SB in the current study. For example, one study found that university students spent an average of 46.6 min per night using electronic media in bed before sleep [55]. Considering the possible SB spent in bed, the total SB of students could be much higher in reality.

The positive relationship between class duration and MVPA was unexpected. One possible explanation is the commute to or movement around campus carried out by fast walking or cycling. Because the methods of detecting MVPA and cycling are different (MVPA identified by a cadence of ≥ 100 steps/minute; cycling detected by hip flexion angle), we cannot conclusively attribute the MVPA to the students’ cycling to university. Nonetheless, days with scheduled classes were positively associated with cycling, with students cycling, on average, 4 min more than on days without classes. This is not unusual, as cycling is the most common mode of transportation for students in The Netherlands [56]. In addition, 95.6% of students in the present study reported that they commute either by foot or by bike. One may say that having classes scheduled could encourage students to be active because of the way they commute. However, the 54 s of MVPA associated with every scheduled class hour is overshadowed by the 9.0 additional minutes of SB.

Addressing the longer-than-expected duration spent on SB, there is reason to advocate a more active lifestyle in students. One possibility is by changing the physical environment of the university, as suggested by a focus group of university students [29]. For example, standing desks have been suggested and implemented within classrooms to counteract the prevalence of prolonged SB in students [57]. Theoretically, introducing standing classes may flip the results of the current study, with longer class durations being associated with less SB, thus proving to be an efficient and effective solution to reduce SB. Several pilot studies have found that introducing LPA through the use of standing desks is feasible within classrooms [58,59]. Although physically active education may reduce the students’ SB duration, further activity monitoring research is required to ensure that these active interventions do not result in compensatory behaviors such as more SB outside the classroom.

In the end, after controlling for class effects, there were still significant day-to-day differences for SB, active SB ratio, LPA, time in bed, and seated transportation, although the effect sizes ranged from small to medium (*d* = 0.06 to 0.30). The reason for these differences can only be speculated on at this point, presenting an interesting avenue for future studies.

The strength of this study comes from the data collection. The large sample spanned all faculties of the university, yielding a representative sample of the student population. In addition, the data collection was carried out throughout the year, covering the students’ year-round pattern of physical behavior. Previous studies have shown that seasons and weather tend to affect one’s PA, especially active transportation during poor or extreme conditions [60,61]. However, we do not expect any structural differences in weather at a day-to-day level that may affect the students’ PA, SB, and choice of transportation across the entire year.

In terms of limitations, the activPAL3™ measures limb position, therefore potentially overlooking physical activities carried out with seated or lying positions (e.g., rowing, swimming, or weight lifting) and overestimating total SB. In the present study, the participants were asked to complete an online diary that was meant to serve as a check for the students’ activities. This daily diary could have confirmed the students’ actual activities, including the possibility that SB was reported as time in bed. However, compliancy of logging the daily diary was very low, despite the current study using an electronic diary, a solution suggested by Edwardson et al. after observing a similarly low compliancy in other studies [31]. Instead of daily diaries, future studies could utilize multiple activity monitors on different limbs to minimize the limitations of tracking activity from one limb. This multi-unit monitoring method is common in clinical settings, but requires further validation work for use in field settings [32].

A recommendation that we have for future research is to consider using a compositional data analysis of the activity variables that constitute a 24-h day [62]. Analyzing each activity separately, as we have done in the current study, does not take into account the interdependence that is inherent in these activities. Importantly, the change in one behavior may be accompanied by an asymmetrical change in one or more other activities. For example, a one-minute increase in SB may not concur with a proportional decrease in the other activities, but may lead to a larger decrease in LPA and time in bed, and a smaller decrease in MVPA. The inclusion of all activities that make up the 24-h day within one multivariate analysis would better illustrate the fluid changes between these activities.

## 5. Conclusions

Attending classes was found to be associated with the students’ day-to-day activity patterns. Importantly, students who attend classes for longer durations engaged in longer bouts of SB. Addressing the concern that the students were found to be leading more sedentary lifestyles than previously expected, actions are required to avoid any further increment in SB. Adapting the academic environment, whether by introducing physically active education or breaks during education, can potentially promote a more active lifestyle. Nevertheless, any intervention requires thorough research to ensure that it balances academic endeavors with physical wellbeing.

## Figures and Tables

**Table 1:** Sample characteristics (*N* = 317).

	***M***	***SD***
Age	19.6	1.4
Height (m)	1.73	0.09
Weight (kg)	65.5	10.3
Body mass index (BMI; kg/m^2^)	21.9	2.7
Travel duration to university (min)	18.6	21.7
Self-reported sleep duration (hours)	7.5	3.7
	**Frequency**	**Percentage (%)**
**Gender**		
Female	220	69.4
Male	95	30.0
Gender Variant/Non-conforming	1	0.3
Prefer not to answer	1	0.3
**BMI (kg/m^2^) [48]**		
Underweight (<18.5)	20	6.3
Healthy (18.5–24.9)	266	83.9
Overweight (25–29.9)	27	8.5
Obese (≥30)	4	1.3
Commuting to class by foot or bike	303	95.6
Having part-time job(s)	73	23.0
**Residential Area [49]**		
Urban	263	83.0
Rural	18	5.6
Not applicable/Living outside The Netherlands	36	11.4
**Faculties**		
Health, Medicine and Life Sciences	154	48.6
School of Business and Economics	61	19.2
Science and Engineering	48	15.1
Law	31	9.8
Psychology and Neuroscience	16	5.1
Arts and Social Sciences	7	2.2
**Living Situation**		
Alone	89	28.1
Parents	49	15.5
Siblings (without parents)	3	0.9
Friends	169	53.3
Partners	7	2.2
**Parent Education Attainment**		
Not applicable	8	2.5
Secondary education	40	12.6
Tertiary education and above	269	84.9
**Gym/Sports Club Membership**		
No	94	29.7
Yes, but inactive	31	9.7
Yes	192	60.6
**Smoking Status**		
Never	237	74.8
Stopped >6 months	19	6.0
Stopped <6 months	14	4.4
Yes	47	14.8
**Alcohol consumption**		
Never	34	10.7
Special occasions	91	28.7
Weekends	69	21.8
Once a week	72	22.7
3–5 times a week	49	15.5
Everyday	2	0.6

**Table 2:** Students’ class duration, physical activity behavior, and type of transportation for each weekday.

		Mon	Tue	Wed	Thu	Fri
Class duration (hours:minutes)	M	2:53	2:16	2:05	2:33	1:35
	SD	1:37	1:41	1:36	1:24	1:19
	N	317	317	317	317	314
SB (hours:minutes)	M	8:47	8:47	8:58	8:40	8:20
	SD	2:21	2:22	2:18	2:39	2:25
	N	317	317	317	317	314
Active SB ratio	M	0.437	0.438	0.434	0.448	0.486
	SD	0.196	0.194	0.189	0.206	0.203
	N	317	317	317	317	314
LPA (hours:minutes)	M	4:14	4:18	4:30	4:33	4:52
	SD	1:42	1:42	1:44	1:55	2:04
	N	317	317	317	317	314
MVPA (hours:minutes)	M	0:28	0:26	0:28	0:28	0:30
	SD	0:25	0:25	0:23	0:25	0:26
	N	317	317	317	317	314
Time in bed (hours:minutes)	M	10:28	10:23	10:02	10:16	10:12
	SD	2:33	2:26	2:25	2:49	2:39
	N	317	317	317	317	314
Cycling (hours:minutes)	M	0:17	0:18	0:19	0:17	0:16
	SD	0:21	0:19	0:20	0:18	0:20
	N	317	317	317	317	314
Seated transportation (hours:minutes)	M	0:24	0:21	0:25	0:25	0:36
	SD	0:52	0:54	0:49	0:52	1:00
	N	317	317	317	317	314

*Note*. M = mean. SD = standard deviation. N = Sample size.

**Table 3:** Association between class duration and students’ activity levels. ^A.^

	Class Duration (Hour)		
Outcome	Model Coefficients	95% CI	*p*-Value
SB (min)	9.0	4.9, 13.1	<0.001
Active SB ratio	−0.011	−0.016, −0.006	<0.001
LPA (min)	1.2	−1.6, 4.0	0.413
MVPA (min)	0.9	0.2, 1.6	0.010
Time in bed (min)	−12.2	−16.6, −7.8	<0.001

*Note*. SB = sedentary behavior. LPA = light physical activity. MVPA = moderate-to-vigorous physical activity. 95% CI = 95% confidence interval. Min = minutes. ^A^ Effects of potential covariates gender and body mass index (BMI) were controlled for within each model. As one participant reported being gender non-conforming, and one other participant preferred not to report their gender, these two participants were removed from the analyses to control for the effects of gender.

**Table 4:** Association between days with classes and students’ method of transportation. ^A.^

	Days with Class(es) ^B^		
Outcome	Model Coefficients	95% CI	*p*-Value
Cycling time (min)	4.0	1.9, 6.0	<0.001
Seated transportation (min)	1.7	−3.8, 7.1	0.549

*Note*. 95% CI = 95% confidence interval. Min = minutes. ^A^ Effects of potential covariates gender and body mass index (BMI) were controlled for within each model. As one participant reported being gender non-conforming, and one other participant preferred not to report their gender, these two participants were removed from the analyses to control for the effects of gender; ^B^ Compared against days with no classes.

## Appendix A

**Table A1:** Pearson correlation between SB, LPA, MVPA, and time in bed across each weekday.

Day	Activities	LPA	MVPA	Time in Bed
Monday	SB	*r* = −0.23 **	*r* = −0.07	*r* = −0.73 **
	LPA		*r* = 0.14 *	*r* = −0.45 **
	MVPA			*r* = −0.20 **
Tuesday	SB	*r* = −0.34 **	*r* = −0.15 **	*r* = −0.67 **
	LPA		*r* = 0.20 **	*r* = −0.37 **
	MVPA			*r* = −0.14 *
Wednesday	SB	*r* = −0.34 **	*r* = −0.08	*r* = −0.71 **
	LPA		*r* = 0.09	*r* = −0.39 **
	MVPA			*r* = −0.16 **
Thursday	SB	*r* = −0.31 **	*r* = −0.05	*r* = −0.70 **
	LPA		*r* = 0.01	*r* = −0.39 **
	MVPA			*r* = −0.10
Friday	SB	*r* = −0.33 **	*r* = −0.03	*r* = −0.63 **
	LPA		*r* = 0.11	*r* = −0.49 **
	MVPA			*r* = −0.21 **

*Note*. SB = sedentary behavior. LPA = light physical activity. MVPA = moderate-to-vigorous physical activity. ** Significant at the 0.01 level (2-tailed); * Significant at the 0.05 level (2-tailed).

**Table A2:** AIC and BIC values indicating model goodness-of-fit.

Activities	AIC	BIC
**SB**		
Model with weekday × class duration interaction	19,736.49	19,880.93
Model with no interaction	19,729.14	19,852.18
**Active SB ratio**		
Model with weekday × class duration interaction	−856.02	−711.58
Model with no interaction	−860.29	−737.24
**LPA**		
Model with weekday × class duration interaction	18,741.23	18,885.68
Model with no interaction	18,735.75	18,858.80
**MVPA**		
Model with weekday × class duration interaction	14,173.21	14,317.66
Model with no interaction	14,168.95	14,291.99
**Time in bed**		
Model with weekday × class duration interaction	19,958.20	20,102.64
Model with no interaction	19,952.46	20,075.51
**Cycling**		
Model with weekday × class interaction	13,290.15	13,434.60
Model with no interaction	13,284.82	13,407.86
**Seated−transportation**		
Model with weekday × class interaction	16,373.56	16,518.01
Model with no interaction	16,368.08	16,491.13

*Note.* SB = sedentary behavior. LPA = light physical activity. MVPA = moderate-to-vigorous physical activity. AIC = Akaike’s Information. BIC = Schwarz’s Bayesian Criteria.

**Table A3:** Pairwise comparisons of significant estimated mean differences in SB (min) between weekdays ^A^.

Day (I)	Day (J)	Estimated Mean Difference (I-J)	95% CI	*p*-Value	Cohen’s *d*
Wed	Thu	21.5	1.5, 41.6	0.036	0.10
	Fri	29.3	9.8, 48.7	0.003	0.21

*Note*. SB = sedentary behavior. 95% CI = 95% confidence interval. ^A^ Estimated mean differences were corrected for class duration, gender, and body mass index (BMI).

**Table A4:** Pairwise comparisons of significant estimated mean differences in active SB ratio between weekdays ^A^.

Day (I)	Day (J)	Estimated Mean Difference (I-J)	95% CI	*p*-Value	Cohen’s *d*
Fri	Mon	0.035	0.008, 0.061	0.011	0.22
	Tue	0.042	0.016, 0.068	0.001	0.21
	Wed	0.048	0.023, 0.074	<0.001	0.23
	Thu	0.029	0.001, 0.056	0.043	0.15

*Note*. SB = sedentary behavior. 95% CI = 95% confidence interval. ^A^ Estimated mean differences were corrected for class duration, gender, and body mass index (BMI).

**Table A5:** Pairwise comparisons of significant estimated mean differences in LPA (min) between weekdays ^A^.

Day (I)	Day (J)	Estimated Mean Difference (I-J)	95% CI	*p*-Value	Cohen’s *d*
Mon	Wed	−17.1	−29.6, −4.5	0.008	0.15
	Thu	−19.4	−32.8, −5.9	0.005	0.16
	Fri	−40.2	−54.7, −25.8	<0.001	0.30
Fri	Tue	33.5	18.3, 48.6	<0.001	0.24
	Wed	23.2	8.4, 38.0	0.002	0.16
	Thu	20.9	5.0, 36.8	0.010	0.13

*Note*. LPA = Light physical activity. 95% CI = 95% confidence interval. ^A^ Estimated mean differences were corrected for class duration, gender, and body mass index (BMI).

**Table A6:** Pairwise comparisons of significant estimated mean differences in time in bed (minutes) between weekdays ^A^.

Day (I)	Day (J)	Estimated Mean Difference (I-J)	95% CI	*p*-Value	Cohen’s *d*
Mon	Wed	32.5	13.0, 52.1	0.001	0.15
	Fri	32.4	9.7, 55.1	0.005	0.08
Tue	Wed	23.4	4.3, 42.6	0.017	0.12
	Fri	23.3	2.2, 44.3	.031	0.06

*Note*. ^A^ Estimated mean differences were corrected for class duration, gender, and body mass index (BMI).

**Table A7:** Pairwise comparisons of significant estimated mean differences in seated transport (minutes) between weekdays ^A^.

Day (I)	Day (J)	Estimated Mean Difference (I-J)	95% CI	*p*-Value	Cohen’s *d*
Fri	Mon	12.5	4.6, 20.5	0.002	0.17
	Tue	15.6	7.8, 23.4	<0.001	0.21
	Wed	12.3	4.8, 19.7	0.001	0.17
	Thu	13.2	5.9, 20.6	<0.001	0.17

*Note*. ^A^ Estimated mean differences were corrected for class effect, gender, and body mass index (BMI).
